# Vitamin C Deficiency in the Young Brain—Findings from Experimental Animal Models †

**DOI:** 10.3390/nu13051685

**Published:** 2021-05-15

**Authors:** Pernille Tveden-Nyborg

**Affiliations:** Section of Experimental Animal Models, Faculty of Health and Medical Sciences, University of Copenhagen, 1870 Copenhagen, Denmark; ptn@sund.ku.dk

**Keywords:** vitamin C, deficiency, brain, development

## Abstract

Severe and long-term vitamin C deficiency can lead to fatal scurvy, which is fortunately considered rare today. However, a moderate state of vitamin C (vitC) deficiency (hypovitaminosis C)—defined as a plasma concentration below 23 μM—is estimated to affect up to 10% of the population in the Western world, albeit clinical hallmarks in addition to scurvy have not been linked to vitC deficiency. The brain maintains a high vitC content and uniquely high levels during deficiency, supporting vitC’s importance in the brain. Actions include both antioxidant and co-factor functions, rendering vitamin C deficiency likely to affect several targets in the brain, and it could be particularly significant during development where a high cellular metabolism and an immature antioxidant system might increase sensitivity. However, investigations of a non-scorbutic state of vitC deficiency and effects on the developing young brain are scarce. This narrative review provides a comprehensive overview of the complex mechanisms that regulate vitC homeostasis in vivo and in the brain in particular. Functions of vitC in the brain and the potential consequences of deficiency during brain development are highlighted, based primarily on findings from experimental animal models. Perspectives for future investigations of vitC are outlined.

## 1. Introduction

Most animals are able to synthesize vitamin C (vitC) in the liver, but a few species including fish, birds, humans, non-human primates, guinea pigs and bats evolved to depend entirely on an adequate dietary vitC supply to ensure survival [1,2,3,4,5]. The disruption of an endogenous hepatic vitC production is due to a mutation in the L-gulono-γ-lactone oxidase gene, which in primates and guinea pigs occurred an estimated 60 and 14 million years ago [6,7,8,9]. The mutation renders vitC biosynthesis dysfunctional and introduces the risk of life threatening vitC deficiency if dietary supplies are scarce.

Recommendations for vitC intake in humans (Recommended Daily Intake, RDI) are primarily targeted to prevent scurvy and differ between countries, ranging between 40 and 110 mg/day for adults, leading to plasma concentrations of 25–60 μM [10,11,12]. Despite recommendations, vitC deficiency/hypovitaminosis C—defined as a plasma concentration below 23 μM (<11 μM denotes severe vitC deficiency) [13]—is estimated to affect up to 10% of the population in the Western world, with increased prevalence in some groups—e.g., smokers, mothers-to-be and children [14,15,16,17,18,19] (reviewed in [20,21]). Large population studies have associated vitC deficiency with increased disease risk such as cardiovascular disease, cancer and metabolic diseases such as type 2 diabetes and fatty liver disease; however, in addition to scurvy, diagnostic hallmarks of vitC deficiency have not been identified [22].

The brain is particularly interesting when considering vitC. The brain has high levels of vitC and is able to maintain levels up to 100-fold higher than other organs during deficiency, emphasizing a preference for vitC in the brain [23,24]. Early symptoms of scurvy include unspecific mood disorders and depression, supporting a negative effect of progressing vitC deficiency on the brain [25]. In addition, mice born devoid of active vitC transport to the brain display cerebral bleedings and do not survive long after birth, demonstrating vitC depletion as detrimental to the brain and perinatal survival [26,27]. VitC depletion (no intake of vitC) in weanling guinea pigs induced oxidative stress and DNA repair markers in the brain, supporting that the newborn brain is sensitive to reductions in vitC, possibly exacerbated by the high growth rate and relatively immature antioxidant system during early life [24]. Thus, the brain constitutes a target organ for vitC and the young brain may be particularly vulnerable to states of deficiency. Putative effects on brain development and function may therefore represent an undetailed and unrecognized consequence of hypovitaminosis C also in humans.

This narrative review is based on selected literature derived from online searches through Pubmed, Web of Science and Google Scholar, applying a topical approach to identify central findings within the scientific field. Keywords included, but were not limited to: vitamin C, ascorbic acid, ascorbate, deficiency, brain and brain development.

## 2. Vitamin C Regulation In Vivo

VitC uptake, distribution and excretion in the body is regulated by different transport mechanisms, which together with a cellular capacity for intracellular recycling leads to a very complex distribution profile [28]. To provide insight into the challenges involved when performing and evaluating studies of vitC, the mechanisms governing vitC homeostasis in vivo are detailed below.

### 2.1. Cellular Vitamin C Uptake

In healthy individuals, the water soluble vitC is predominantly present as the ascorbate anion (ASC) with only a negligible proportion in the oxidized form as dehydroascorbic acid (DHA) [29]. As most cells effectively recycle ASC from DHA, both are generally considered as contributors to the total vitC pool.

#### 2.1.1. Ascorbic Acid Transport

The primary transport of vitC is achieved through active transport of ASC. It is governed by the membrane bound sodium-dependent vitC co-transporters (SVCTs), enabling an increased concentration of ASC in cells and tissues through an energy demanding and sodium coupled co-transport [28,30,31,32]. Though there are exceptions, e.g., erythrocytes and astrocytes, that do not express SVCTs and rely on simple diffusion of ASC and facilitated DHA diffusion [33,34], SVCT-mediated transport is considered the predominant regulator of vitC transport in vivo. The SVCTs consists of two types, the SVCT1 and SVCT2 transporters (encoded by the *SLC23A1* and *2* genes, respectively), not uniformly present in tissues and cells [32,35].

Based on distribution and chemical properties (affinity and capacity), the SVCT1 is considered the main transporter involved in intestinal uptake and renal re-uptake, thereby regulating systemic vitC homeostasis, whereas the SVCT2 transporter governs the ASC transport from blood and extracellular fluids to tissues [28,34,36]. The SVCT-1 resides predominantly in epithelioid cells and is characterized by low affinity (Km of 65–252 μM) and high capacity (Vmax around 15 pmol/min/cell) transport, supporting the role as “bulk-transporter” of ASC [30,37,38]. For example, the SVCT1 transporter is located on the apical side of the intestinal epithelia surface cells enabling ASC uptake from the gut luminal content and in kidney tubular epithelia permitting the re-uptake of ASC from the glomerular ultra-filtrate [32,39].

The SVCT2 transporter is expressed in most cell types and has a higher affinity (Km of 8–69 μM) but lower capacity (Vmax around 1 pmol/min/cell) compared to the SVCT1, supporting a role in maintaining cellular ASC levels even when extracellular concentrations are low [30,35,40]. Two additional SVCTs have been characterized (SVCT3/*SLC23A3* and SVCT4/*SLC23A4*), but they have not been shown to be involved in vitC transport [41,42]. For both SVCT1 and two single nucleotide polymorphisms have been identified potentially affecting transporter capacity; however, data on the putative effects on vitC kinetics and the distribution of polymorphisms within populations is currently lacking [22,43,44].

#### 2.1.2. Dehydroascorbic Acid Transport

Extracellular DHA levels maintains a concentration gradient across the cell membrane that enable passive diffusion from plasma and from the extracellular fluid to the cell [28]. Calculations of the recycling capacity of erythrocytes have estimated that the total amount of ASC in the blood stream can be recycled within 3 min, underlining an important role of red blood cells in maintaining circulating redox balance and—potentially—also serving as an ASC reservoir [45,46]. Generally, the DHA concentration in the blood stream is much lower than that of ASC, and the contribution to the cellular vitC pool is therefore often considered as largely negligible. However, in cases of increased DHA concentrations, for example, during systemic oxidative stress and in states of inflammation, the contribution of DHA may be higher [31].

In addition to simple diffusion across the cell membrane, DHA uptake occurs by facilitated diffusion through membrane bound glucose transporters (GLUT 1–4 and 8) present in several cell types in the body [47,48,49,50,51]. Some cells, such as erythrocytes and astrocytes, only express GLUT transporters and therefore rely exclusively on DHA uptake as a vitC source [33,34]. DHA essentially competes with glucose in GLUT-mediated transport. By principle, facilitated diffusion allows for a concentration-driven bi-directional transport through GLUT transporters, but the intracellular reduction of DHA to ASC favors a continued DHA diffusion into the cell, while it does not allow for an active increase in concentration of intracellular DHA levels.

**Figure 1:**
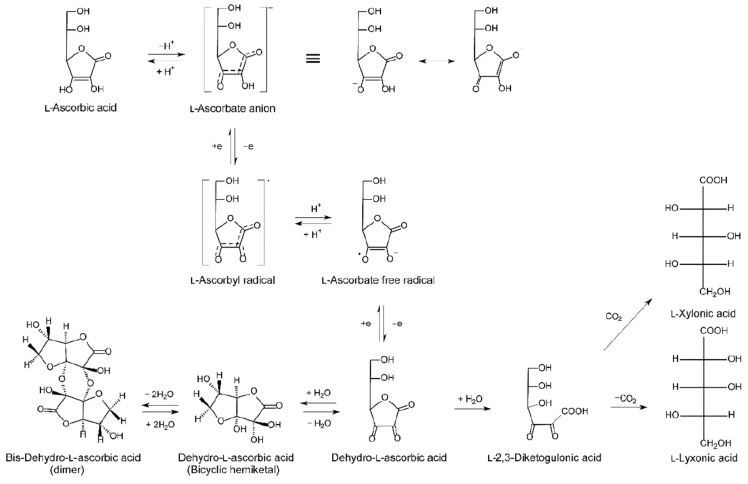
Schematic outline of ascorbic acid oxidation and nomenclature. Ascorbate (ASC) readily quenches free radicals by donating an electron. thereby forming the ascorbate free radical with a half-life ranging from 10^−3^ s to minutes. The ascorbate free radical can be reduced back to ASC or subjected to further oxidation and produce dehydroascorbic acid (DHA). In turn, DHA may be hydrolyzed, irreversibly altering the molecular structure to 2,3-diketogulonic acid with no vitamin C activity, and proceed to be metabolized and cleared [34]. Alternatively, DHA is reduced by glutathione (GSH) to form the ascorbate free radical and subsequently ASC, or even directly to ASC by enzymatic reaction [34,52,53] (The figure is reproduced without modifications from [28] and in accordance with CC by 4.0).

Intracellular recycling constitutes a cornerstone in vitC homeostasis. Fulfilling the role as one of the most efficient low-molecular weight antioxidants, ASC readily donates an electron to quench free radicals, consequently becoming oxidized and leading to DHA formation [29]. DHA can then either be recycled back to ASC or metabolized [34,52,53] (Figure 1). When extracellular DHA is absorbed to the cell, it is immediately reduced to ASC, thereby promoting equilibrium-driven DHA absorption. In this way, cellular concentrations of ASC and DHA can be balanced as long as the necessary reducing agents are available.

### 2.2. Cellular Vitamin C Efflux

Albeit essential in maintaining whole body homeostasis, the transport mechanisms of vitC out of cells is surprisingly undisclosed. The SVCTs appear to serve only as influx transporters and are not actively engaged in ASC efflux [54]. Passive diffusion is expected; however, this route is only relevant for the <1% unionized ASC fraction at a physiologically neutral pH [28]. In erythrocytes, intracellular reduction in DHA promotes ASC release but at a low rate of around 10% compared to the DHA uptake [55], and both hepatocytes and endothelial cells have been shown to release ASC in response to intracellular ASC accumulation at a faster rate than is permitted by simple diffusion [56,57].

Thus, it seems that other routes of outward transport must take part in vitC regulation. This is also clear in view of a maximum plasma concentration occurring around 3 h after an oral administration of 250 mg ascorbic acid in humans, unlikely to be achieved by simple diffusion across the basolateral membrane of intestinal epithelial cells and into the blood stream [58].

Membrane bound anion channels for ASC efflux have been suggested; however, their contribution to vitC homeostasis in vivo has not been determined [31,54,59]. Osmotic swelling of astrocytes in vitro induces ASC release, suggesting osmoregulation and volume-sensitive anion channels as a mechanism of providing extracellular ASC—e.g., for uptake in neurons [31,60]. Likewise, studies in cultured neuroblastoma cells suggest that anion channels are involved in neuronal ASC efflux in response to glutamate [61]. In vitro studies of human pericytes suggests alternative efflux systems in these cells. The existence of one or more undisclosed vitC efflux mechanisms is substantiated by the ability of certain cell types to secrete vitC in response to endogenous signaling, such as the rapid (within minutes) release by adrenal glands in response to an intravenous dose of adrenocorticotrophic hormone [62]. Additionally, ASC efflux in the brain is induced by the neurotransmitter glutamate, linking neuronal signal transduction to ASC efflux and vitC homeostasis [63,64].

Collectively, these findings strongly support the presence of more than one mechanism to regulate ASC efflux, some with distinct tissue or cell specificity. However, though the release of intracellular ASC to the extracellular environment is indisputable, there are several unknown characteristics of the regulation of vitC homeostasis ultimately limiting our ability to predict and interpret results.

### 2.3. Vitamin C Pharmacokinetics

**Figure 2:**
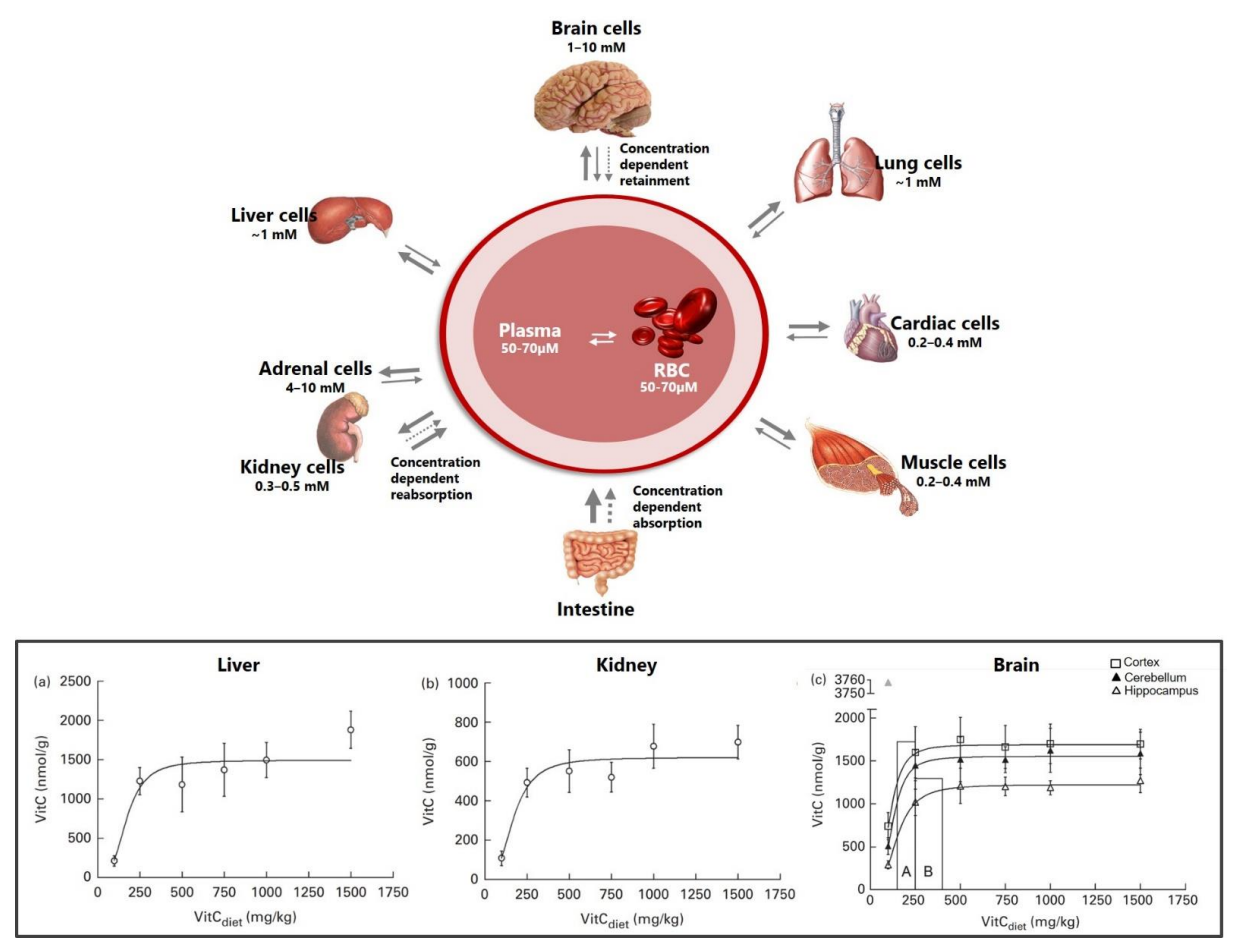
The distribution of vitamin C in the body. Distribution of vitamin C (vitC) in vivo is highly differential. Some organs have concentration-dependent mechanisms for retention of vitamin C, maintaining high levels during times of inadequate supply at the expense of other organs. In addition, the concentration-dependent absorption and re-absorption mechanisms contribute to the homeostatic control of vitC in the body. The brain upholds relatively high levels compared to other organs, with neurons displaying up to 10 mM. Inserted graphs show the dose–concentration curves measured in guinea pigs subjected to different dietary vitC doses, with estimated curve fitting (Hill equation); (**a**) liver, (**b**) kidney and (**c**) brain with cortex, cerebellum and hippocampus levels depicted individually. In the brain, the hippocampus achieves saturation (A) at a higher dose, but with a smaller concentration maximum (Cmax) compared to cortex and cerebellum (B), illustrating a regional difference in vitC distribution within the brain. In the liver and kidney, saturation is not as clear, suggesting a more direct reflection of the increasing plasma concentration compared to the brain. This supports that vitC transport to the brain is different from that of other organs and allows for the brain to be favored in vitC distribution. Moreover, the dose–concentration relationship underlines that accurate tissue levels of vitC are difficult to extrapolate from plasma levels. (Reproduced and modified from [28,73] and in accordance with CC by 4.0.).

Regulated primarily by membrane bound transporters, vitC uptake and excretion is a saturable process and follows a dose-dependent non-linear pharmacokinetic profile [28]. In healthy humans subjected to increasing doses of vitC, plasma concentrations plateau at 70–80 μM (steady-state) at a daily intake of 200–300 mg vitC [11,12,65]. Tissue levels (at steady state) range from 0.3 mM in muscles and 1 mM in the liver, and up to 10 times that in the brain neurons and adrenal glands, which have the highest levels in the body [28,30,66] (Figure 2). The preferential increase in concentration of vitC in tissues depends on the specific cell type (expression of transporters) and vitC concentration. In this aspect, the brain and adrenal glands distinguish themselves by the ability to maintain high vitC levels, even during chronic states of deficiency and depletion [23]. Data from guinea pigs show that vitC depletion (no vitC in the diet and leading to a prescorbutic state) reduced liver levels 60–100-fold to around 26 nmol/g tissue and kidney levels more than 50-fold to around 13 nmol/g tissue within 21 days [24,67]. The brain, however, maintained vitC levels of 500–300 nmol/g tissue (varying on brain region), corresponding to about 1/3 of non-depleted levels and despite very low vitC plasma concentrations of 1 and only 3 μM in the cerebrospinal fluid (CSF) [24,67]. Though overall levels differ from those reported in guinea pigs, the ability to retain vitC in the brain during states of deficiency and depletion is conserved in *gulo*^−/−^ mice unable to synthesize vitC [68,69]. Compromised vitC synthesis has also been shown in aldehyde reductase 1a deficient (*akr1a^−/−^*) and senescence marker protein 30/glucolactonase knock-out (SMP30/GNL^−/−^) mice strains, which are also applied in vitC research [70,71,72].

In guinea pigs subjected to different levels of dietary vitC, saturation was achieved in most tissues by doses of 500 mg vitC/kg feed and a plasma concentration of 40 μM (±17.6 SD) [73]. Notably, the dose–concentration curves for the brain and adrenal glands showed earlier saturation. Adrenal glands reached a plateau at 250 mgvitC/kg feed, and in the brain the frontal cortex and cerebellum reached a plateau at 150–250 mgvitC/kg feed and the hippocampus at 250–400 mgvitC/kg feed [73] (Figure 2). The individual data points indicate that the liver and kidney continue to reflect increasing plasma concentrations—though at a slower rate—whereas the brain and adrenal glands do not exhibit this to the same degree. Moreover, the brain and adrenal glands are readily repleted once a higher level of vitC is introduced, suggesting a high prioritization of these organs [28,73,74]. Together, this underlines the highly complex and differential distribution of vitC to tissues at different degrees of saturation. Importantly, the data also show that plasma concentrations alone do not directly predict tissue concentrations.

## 3. Vitamin C Transport to the Brain

The brain has unique qualities in relation to vitC transport and distribution, with neurons displaying very high levels of vitC (up to 10 mM) at plasma concentrations of 50–70 μM [75,76]. Moreover, the brain maintains impressively high vitC levels during deficiency, even when most other organs are depleted [24,73]. Studies in guinea pigs show that during an absence of dietary vitC (depletion), the liver and kidneys are depleted within 3–4 weeks, levels dropping to less than 2% of control levels [24,67]. The brain, however, maintains levels of around 25–30% (3–500 nmol/g vitC) of controls even at a plasma concentration of 1 μM and the appearance of early clinical symptoms of scurvy (weight stagnation) [67]. Following a persistently low, but non-scorbutic, intake of vitC (100 mgvitC/kg feed), leading to a plasma concentration of 4–5 μM, the brain maintains levels between 100–150 times higher than plasma, compared to a 15–20-fold increase when plasma concentrations are 70 μM [67,73]. The ability to favor vitC levels even during prolonged states of vitC deficiency in the brain is conserved across age groups from early life to young, mature and old guinea pigs [24,74,77,78,79]. This emphasizes a high prioritization of the brain and supports that vitC is pivotal in the brain through all life phases. However, the mechanisms governing this preferential transport and retention of brain vitC levels remain incompletely understood.

### 3.1. Crossing the Blood–Brain Barrier

With few exceptions, a molecule must pass through the blood–brain barrier (BBB) or through the choroid plexus into the CSF to reach the extracellular space and brain tissue, thereby forming a restrictive barrier for passage to the brain [80].

#### 3.1.1. ASC Transport

The primary transport of vitC to the brain is through the SVCT2 transporter, whereas the SVCT1 is absent [32,38]. Studies in knock-out mice devoid of the SVCT2 transporter (*svct2*^−/−^ or *slc23a2*^−/−^) have demonstrated an essential role of SVCT2-mediated vitC transport to the brain [26,27]. Fetal development appears normal at term but offspring numbers are reduced. Levels of vitC in the brain and lungs are close to undetectable, confirming the absence of SVCT2 transport, and, once born, pups die almost immediately showing cerebral bleedings in cortical surfaces and deeper brain structures, oxidative stress and apoptosis in the brain, but without signs of generalized scurvy [26,27].

SVCT2 transporters are located in the choroid plexus endothelium and enable the active uptake of ASC from the blood stream [33,81,82,83], and in vivo studies in mice have shown a rapid distribution of 14C-labelled vitC to the choroid plexus upon infusion [84]. In situ hybridization studies have confirmed the presence of SVCT2 in the choroid plexus, supported by in vitro culture studies showing active and Na-dependent transport (Km of 67 μM) as main regulator of ASC uptake, consolidating the SVCT2-mediated transport in the choroid plexus [85]. ASC then crosses the choroid epithelia through diffusion or efflux mechanisms to enter the CSF. SVCT2 immuno-reactivity has revealed expression in the ventricular ependymal cells and tanocytes, suggesting this is a route of ASC transport from the CSF into the brain; however, the extent of this transport has yet to be determined [82,83].

#### 3.1.2. DHA Transport

**Figure 3:**
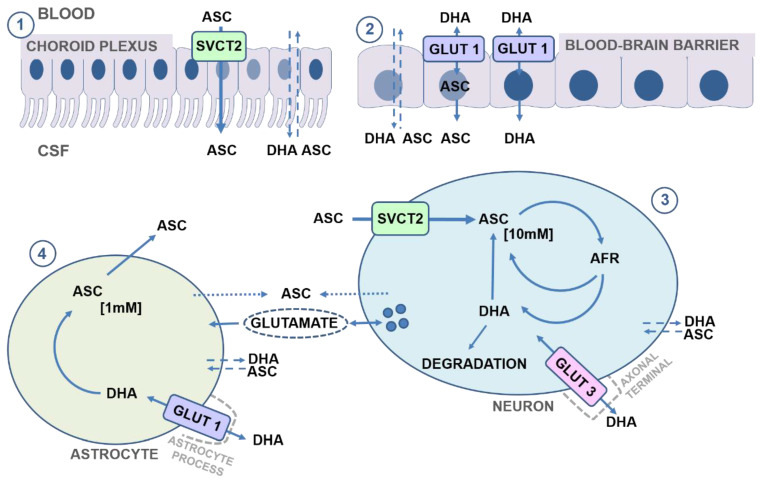
Schematic overview of mechanisms of vitamin C uptake and recycling in the brain. (**1**) Vitamin C (vitC) primarily enters the brain either by SVCT2-mediated ascorbate (ASC) transport through the epithelial cells of the choroid plexus to the cerebrospinal fluid (CSF), or as dehydroascorbic acid (DHA) via glucose transporter 1 (GLUT1) situated on the blood–brain barrier (BBB) endothelia (**2**). DHA may be recycled to ASC within the BBB-endothelial cells or released directly to the extracellular matrix. Passive diffusion of ASC and DHA may also occur; however, efflux mechanisms regulating vitC release are yet largely unaccounted for. (**3**) Extracellular ASC mainly enters neurons through SVCT2 transporters. Intracellularly, ASC may be oxidized leading to formation of the ascorbate free radical (AFR). AFR can then form ASC and DHA. DHA may be recycled to ASC through reduction, be transported out of the neuron e.g., by diffusion, or cleared (degraded). Neurons also possess GLUT3 transporters, allowing for facilitated diffusion of DHA. Together, these mechanisms enable the increase in concentration of high intracellular ASC in neurons, reaching as much as 10 μM. ASC may be released from neurons in response to glutamate uptake. (**4**) Astrocytes do not express SVCT transporters but can take up DHA through GLUT1 facilitated diffusion. DHA is recycled to ASC maintaining a concentration gradient across the astrocyte plasma membrane and promoting the continued DHA uptake and enabling the increase in concentration of intracellular ASC. ASC can then be released from the astrocytes to the extracellular matrix for subsequent uptake to neurons. This can be, e.g., in conjunction with neuronal glutamate release, where glutamate uptake and clearance by astrocytes prompts the release of ASC. AFR: Ascorbate free radical; ASC: Ascorbate; BBB: Blood–brain barrier; CSF: Cerebrospinal fluid; DHA: dehydroascorbic acid; GLUT: glucose transporter; SVCT: sodium coupled vitamin C co-transporter; VitC: vitamin C.

The blood–brain barrier endothelia does not express SVTC2 transporters, but has membrane bound GLUT1 transporters that enable facilitated DHA diffusion [86,87](Figure 3). In the brain microvasculature, GLUT1 is expressed on the luminal side of endothelial cells with increased intensity adjacent to astrocyte processes [88]. The contribution of DHA transport across the BBB to the overall vitC status of the brain is likely negligible in healthy individuals, but it is possible that DHA transport to the brain may increase—for example, in cases of increased oxidation rates during disease [89,90]. However, the extremely low vitC levels, induced brain damages and death of the *svct2*^−/−^ mice effectively underline that DHA transport to the brain in itself is insufficient to maintain adequate vitC levels during states of depletion [26,27].

### 3.2. Inside the Brain

#### 3.2.1. Vitamin C Transport to Neurons

Neurons display some of the highest levels of vitC in the body, reaching up to 10 mM at plasma concentrations of 50–70 μM [75,76] (Figure 3). Neurons express both SVCT2 and GLUT transporters, with the SVCT2 transporter considered as the main source of vitC uptake. In vivo, SVCT2 expression appears to be mainly located to the soma but has also been shown to be extensively expressed in neuronal axons in vitro [32,75,83,91,92,93]. SVCT2 expression differs between brain regions and is most intensive the cerebral cortex, the hippocampus, the dentate gyrus and the cerebellum [83]. VitC levels support SVCT2 as the primary neuronal ASC transporter, reflected by high vitC levels in the cortex (frontal and parietal), cerebellum and the hippocampus in humans, rats, mice and guinea pigs, though absolute levels may differ between species [67,68,73,76,94,95,96]. Dose–concentration curves in guinea pigs show a higher Cmax in the cerebellum and frontal cortex compared to the hippocampus (1689, 1552 and 1223 nmol/g), with saturation of the cerebellum and cortex at a doses of ~200 mg vitC/kg feed compared to the hippocampus (~300 mg vitC/kg feed) [73] (Figure 2). This illustrates that vitC transport to the brain is prioritized between regions and that the hippocampus may be less prioritized during long-term deficiency and perhaps more susceptible to negative consequences of a low vitC intake [73]. Cultured hippocampal neurons from *svct2*^−/−^ mice display reduced growth compared to controls, supporting the essential role of SVCT2 in neuronal development and function [93]. Surprisingly, vitC depletion and deficiency in vivo is not reflected by an upregulation of the mRNA expression of the SVCT2 transporter in brain tissue, implying that other mechanisms may be crucial in maintaining brain vitC levels [67,77,79,97].

In addition to the SVCT2-mediated ASC transport, neurons express GLUT-3 mainly in neuronal processes—i.e., axonal terminals and dendrites in the neuropil—in accordance with high-energy demands such as synaptic activity [33,48,88,98,99,100]. Notably, DHA is potentially toxic to neurons, consuming reducing agents in the recycling process, and GLUT-mediated DHA uptake may therefore potentially exacerbate oxidative stress and associated cell damaging effects [101,102]. GLUT-3 immunoreactivity shows a differential expression pattern in neonatal infants compared to adults, indicating a potential maturation of transporter mechanisms during development and maturation of the brain [99].

During embryonic development, ASC levels differ between brain regions and over time (embryonic day (E) 15–18 in mice), likely due to increased requirements [97]. SVCT2 expression did not cause increases in ASC in the cortex and cerebellum in mice but increased with developmental stage from around E13 (neurogenic period) toward the gliogenic period (E15–19) and during early postnatal life [97,103]. SVCT2 expression in the embryonic neuroepithelia of the ventricular and subventricular zones, and in the embryonic choroid plexus cells, further supports that ASC transport to the CSF, and subsequently neuronal and glial precursor cells, is important for cellular differentiation during early development [92]. In developing mice, SVCT2 mRNA and protein expression displayed an inverse expression pattern to ASC levels, with a marked postnatal increase in the cortex and cerebellum, suggesting changes in brain ASC requirements and SVCT2 distribution during development [97,104]. In postnatal mice pups, the distribution of SVCT2 mRNA differed within cortical and cerebellar regions over time, due to the sequential maturation of neurons and synapses, and opposed to a more uniform distribution in the adult brain [104,105].

#### 3.2.2. Vitamin C Transport to Neuroglia

Non-neuronal cells/glia in the brain do not express SVCT2 in vivo and rely on GLUT-mediated DHA transport through GLUT-1, with astrocytes currently being the most investigated [81,106,107]. GLUT-1 expression is reported in astrocyte processes within the neuropil and in astrocyte foot processes in close proximity to the vasculature [88,100]. Astrocytes possess high reducing competences compared to neurons and DHA is rapidly reduced, enabling intracellular increase in concentration of ASC with vitC levels reaching around 1 mM [33,75] (Figure 3). The ability to accumulate high vitC levels by releasing DHA for GLUT-mediated uptake in neighboring cells, subsequent reduction and ASC accumulation (“bystander effect”) has been reported for other cell types and proposed as a model for neuronal–glial interplay to regulate vitC homeostasis in the brain [87,108,109]. Through GLUT-1-mediated transport, astrocytes take up DHA, recycle it to ASC and sequester ASC intracellularly for release to the extracellular matrix (ECM) and subsequent neuronal uptake. In this way, DHA is effectively recycled through cellular compartmentalization, highlighting astrocytes as an important ASC source [87]. How ASC is released from astrocytes to the ECM is not clear, but volume and ion regulated channel mechanisms as well as a potential coupling to the glutamate release and re-uptake exchange system have been proposed by in vitro studies [60,63,110,111].

It is possible that the astrocyte “ASC reservoir” can provide additional protection against oxidative damage to the brain. This could, for example, be true in cases of neuronal hypoxia, allowing for fast uptake and recycling of excess DHA and, in turn, the release of ASC. Hypoxia induced SVCT2 protein in brain capillary endothelia [112,113] and increased SVCT2 mRNA expression in neurons and in astrocytes surrounding core lesions, underlining hypoxia as an inducer of brain ASC transport mechanisms and suggesting that astrocytes may possess the ability to induce ASC transport during hypoxic conditions [81,106,112,113]. A recent study in rats links SVCT2 expression in astrocytes to induced reactive astrogliosis and, potentially, to direct brain trauma, suggesting that astrocyte reactivity may induce changes in ASC transport, at least in certain types of brain disease [114].

## 4. Vitamin C Functions in the Brain

**Figure 4:**
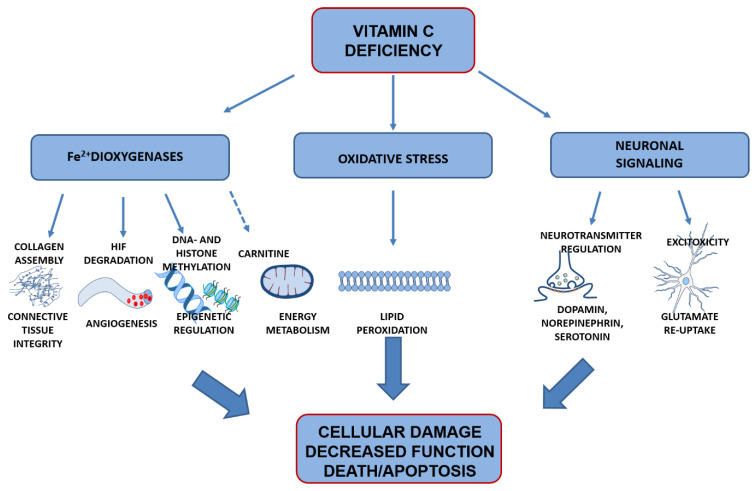
Overview of potential targets of vitamin C deficiency in the brain. Functions of vitC have not yet been completely disclosed and it is linked to several and different roles within the brain. The most well-known role is ensuring the hydroxylation and subsequent assembly of collagen in its triple helical structure. Failure to form functional collagen is seen during long-term and severe vitC deficiency and leads to the breakdown of connective tissue structures, e.g., in vascular walls, hallmarking scurvy. VitC is also linked to the formation of vasculature through hypoxia-inducible factors (HIFs). A lack of vitC may reduce hydroxylation and subsequently accumulation of HIF1α, leading to deviated angiogenesis. In addition, oxidative stress may activate HIFs, thereby increasing levels further. Acting as co-factor in the regulation of methylation of nucleic acids, vitC deficiency is linked to alterations in DNA and histone methylation patterns and subsequent alterations in the epigenetic regulation of gene expression. VitC also acts as co-factor in carnitine synthesis and, though most likely due to alterations in excretion, carnitine deficiency is associated with low vitC status and consequent reductions in mitochondrial fatty acid metabolism, compromising cellular energy metabolism. In turn, accumulating reactive oxygen species and oxidative stress in vitC deficiency may lead to peroxidation of cellular membrane lipids, compromising cellular function and viability. Directly associated with neurotransmitter synthesis, vitC is a co-factor in the hydroxylation of dopamine, leading to norepinephrine, and provides reducing equivalents for tetra-hydrobiopterin necessary for the synthesis of dopamine and serotonin. Lastly, vitC deficiency reduces the re-uptake of extracellular glutamate, which in turn may lead to excitotoxic damage in the brain. Together, these functions of vitC highlights several likely effects of states of deficiency with putative serious consequences for cellular health and brain function.

VitC is one of the most efficient low molecular weight antioxidants in biological systems, and all known biological functions are associated with the reducing properties of ASC. Residing low in the one-electron reduction potential (“pecking order”) of free radical reactions, ASC readily donates reducing equivalents to quench free radicals, such as reactive oxygen species (ROS), or restore other antioxidants, such as vitamin E, while becoming oxidized in the process [29,115,116,117]. Parts of normal cellular metabolism ROS are kept at bay by enzymatic and antioxidant reductions, balancing this metabolic by-product to maintain redox homeostasis. If the balance is disturbed, for example, by decreased antioxidant levels, ROS can accumulate to reach adverse levels, generating oxidative stress that can damage cellular membranes, organelles and DNA, and ultimately have detrimental effects on cell function and survival. In addition to the unspecific antioxidant function, vitC has more specific functions by, for example, acting as ca o-factor in enzymatic reactions. The sections below provide an overview of main functions linked to vitC in the brain and which may consequently be susceptible to negative effects in the case of vitC deficiency (Figure 4).

### 4.1. Preventing Oxidation of Poly-Unsaturated Fatty Acids

A key and generalized antioxidant function of ASC in the brain is to preserve membrane integrity and function by preventing ROS from inducing lipid peroxidation [29]. Low vitC directly increases lipid peroxidation even when other antioxidants (vitamin E and glutathione (GSH)) are present, demonstrating a central role of ASC in preventing oxidative damage to cell membranes [118,119]. In this aspect, the brain may have increased sensitivity due to a high metabolic activity combined with high levels of long chained poly-unsaturated fatty acids (PUFAs) prone to oxidation [120,121,122]. Due to an immature antioxidant system and high cellular growth rates, this may be even more important in the developing brain [123].

PUFAs such as docosahexaenoic acid and arachidonic acid are the primary components of neuronal cellular membranes including neuronal synapses [122,124,125]. The composition and integrity of the pre- and postsynaptic membrane is central for neurotransmitter release, receptor binding and degradation, emphasizing that dynamic regulation of the synaptosome lipid membranes is crucial for neuronal signaling (reviewed by [126]). PUFAs and PUFA derivatives are also linked to neuronal signal transmission through the release of mono-amine neurotransmitters, gamma amino-butyric acid (GABA) and glutamate release and re-uptake [120,127,128,129]. Inside the cell, ROS can react with membrane PUFAs yielding fatty acid radicals, lipid peroxyl radicals and lipid peroxide, which can promote additional lipid peroxidation and establishing a self-propagating vicious circle. Oxidation fragmentizes PUFAs into smaller cytotoxic molecules (e.g., malondialdehyde (MDA) and 4-hydroxy-2-nonenal and carboxyalkylpyrrol-protein adducts), damaging the cell and cellular membranes, and associated with decreased neuronal function and neurodegenerative disorders [120,130,131,132,133]. Specifically, during brain development, PUFAs are thought to be important in the regulation of proliferation and survival of neuronal progenitors [134,135,136]. Severe vitC deficiency more than doubled MDA in weanling guinea pigs compared to non-deficient counterparts and in *svct2*^−/−^ mice pups F_2_-isoprostanes and F_4_-neuroprostanes (peroxidation products of arachidonic acid and docosahexaenoic acid, respectively) were significantly increased [24,27]. In agreement with this, findings from *gulo*^−/−^ mice showed increased brain MDA levels, reflecting decreased ASC levels and also indicated regional differences in brain lipid peroxidation [137]. This highlights the essential functions of PUFAs in the brain and supports ASC as a pivotal antioxidant preventing lipid peroxidation, safeguarding neuronal membrane integrity, function and survival.

### 4.2. Co-Factor for Fe^2+^-2-Oxogluterate-Dependent Dioxygenases

#### 4.2.1. Collagen Synthesis

The most well-known function of vitC is its role in collagen formation, enabling the assembly of triple helix collagen by acting as co-factor in the hydroxylation of collagen polypeptides by Fe^2+^-2-oxogluterate-dependent dioxygenases. ASC deficiency results in insufficient hydroxylation and subsequent release of procollagen instead of stable collagen [138,139,140]. The resulting dysfunctional collagen formation ultimately causes a collapse of collagen structures, e.g., in vascular walls, as reflected in the clinical hallmarks of scurvy with petechial bleedings in skin, gingiva and subperiosteally due to capillary frailty [141,142,143,144]. Fetal s*vct2*^−/−^ mice have decreased collagen IV levels in brain basement membranes, but increased levels in parietal endoderm cells, supporting that though cellular synthesis of pro-collagen is intact, the secretion and assembly of mature collagen IV is not [27].

#### 4.2.2. Hypoxia-Inducible Transcription Factors

A role in Fe^2+^-2-oxogluterate-dependent dioxygenase hydroxylation also places ASC in the regulation of hypoxia-inducible transcription factors (HIFs), of which HIF1α is most abundant [145,146]. HIFs regulate the transcription of genes promoting angiogenesis, apoptosis and changes in cellular metabolism in response to decreasing oxygen tension [147,148]. At physiologically normal oxygen levels HIF α-subunits are hydroxylated and destined for degradation, however, in the absence of oxygen hydroxylation is inhibited and HIF α-subunits are activated (stabilized and assembled) [147]. As ROS promotes HIF accumulation ASC also plays an indirect role in HIF regulation through ROS quenching [149]. HIF1α-induced transcription has been linked to increased neuronal cell death and functional deficits following brain trauma and hypoxia-ischemia-induced brain damage [150,151,152]. However, in less severe states of hypoxia HIF-activation leads to increased transcription of neuroprotective genes, such as erythropoietin and vascular endothelial growth factor [153,154]. In the developing brain, hypoxia and subsequent HIF-activation modulate cellular metabolism, proliferation and angiogenesis, ensuring organogenesis and cellular differentiation [155,156]. In this way, HIF-activation and subsequent transcription of target genes represents a ‘double-edged sword’ in which protective and destructive mechanisms can be induced, depending on the concourse and severity of hypoxia. In the event of vitC deficiency, it may be speculated that reduced availability of ASC in the brain decreases HIF degradation and increases ROS, thereby disturbing the balanced transcriptional regulation of potentially critical factors for normal brain development [123].

#### 4.2.3. Epigentic Regulation

VitC has been shown to be involved in the epigenetic regulation of cellular programming through the Fe^2+^-2-oxogluterate-dependent dioxygenases involved in histone demethylation and through the ten-eleven-translocation 1–3 (TET1-3) enzymes, which catalyses the hydroxylation of 5-methylcytosine to 5-hydroxymethylcytosine on DNA CpG-dinucleotides [157,158,159]. Data from human embryonic stem cells show that vitC, in contrast to other antioxidants, has the capacity to enhance TET-enzymatic activity and to alter DNA methylation patterns promoting blastocyst characteristics [160]. In vivo studies of mice pups (E 13.5) derived from vitC deficient *gulo*^−/−^ dams link vitC deficiency to aberrant TET1 activity and subsequently deviated DNA methylation patterns during germ cell development [161]. In addition, maternal VitC depletion reduced 5-hydroxymethylcytosine levels in embryonic brain and liver [161]. Likewise, vitC deficiency in SMP30/GNL^−/−^ offspring caused significant alterations in the DNA methylation status of investigated target genes in the liver [162].

Fetal midbrain stem cells from rats support ASC as key in the differentiation and transcription of genes characteristic of maturation (e.g., nuclear receptor related 1, *Nurr1*) of dopaminergic neurons in culture [163]. ASC increased 5-hydroxymethylcytosine positive cells as well as the production of tyrosine hydroxylase and dopamine in a dose-dependent manner, which was abolished by blockage of the SVCT2 transporter but also by blocking of TET1 [163]. This indicates that vitC is crucial for the development of a dopaminergic phenotype and that the transcriptional regulation is orchestrated—at least in part—through TET1-mediated methylation patterns [163]. In addition, vitC has been shown to regulate histone demethylation (Histone H3 subunit with lysine 27 tri-methylation; H3K27m3) through an increased activity of Jumonji domain-containing protein D3 [163,164].

Together, these findings suggest an important role of vitC-mediated cross-talk in methylation and demethylation patterns during embryonic development and neuronal differentiation, probably achieved through effects on TET-activity and histone demethylation. However, though not unlikely to play a regulatory role, a direct association between vitC deficiency and disparities in DNA methylation with consequences for brain development and function later in life has yet to be established. In addition, the concentration of ASC required to maintain normal TET and methylation-demethylation activity in vivo and how this translates to a daily vitC intake is currently not known.

#### 4.2.4. Carnitine Availability

Carnitine is supplied through the diet as well as synthesized in the liver, kidneys and the brain, where ASC functions as co-factor in two steps involving Fe^2+^-2-oxogluterate-dependent dioxygenases (6-N-*trimethyllysine dioxygenase* and γ-butyrobetaine dioxygenase) [165]. ASC deficiency has previously been linked to reduced carnitine synthesis in scorbutic guinea pigs [166,167] however, it was later shown that increased carnitine excretion rather than reduced synthesis is the most likely cause of the reported reduced levels of carnitine during vitC deficiency [71,168,169]. Disruption of 6-N-*trimethyllysine dioxygenase* or γ-butyrobetaine dioxygenase activity can have serious consequences for brain development and possibly brain function in humans [170,171]. In addition, carnitine supplementation has been shown to improve hypoxia-induced brain oxidative stress and cognitive impairment in adult rats, and in neonatal rats carnitine decreased induced hypoxia/ischemic brain neuronal cell death, oxidative stress and expression of HIF1α [172,173,174]. Acetylated-L-carnitine improved motor- and cognitive outcomes in experimental models of neonatal hypoxia and traumatic brain injury, and decreased levels of acyl-carnitine has been reported in infants suffering from neonatal hypoxic-ischemic encephalopathy, supporting carnitine to play a role in neuroprotection in the brain [175,176,177,178].

In addition, muscle weakness, fatigue and a reluctance to move are hallmarks of scurvy [144]. This could potentially be due to a peripheral effect of decreased carnitine levels in striated muscles, supported by preliminary findings linking vitC deficiency in humans to decreased fatty acid oxidation and fatigue during moderate exercise [179]. A reduction in carnitine tissue levels and an increase in carnitine excretion was shown in scorbutic guinea pigs, however this was not detected in scorbutic mice (SMP30/GNL^−/−^) suggesting that the effects of vitC deficiency on carnitine may not be uniform between species [71].

### 4.3. Signal Transduction

#### 4.3.1. Monoaminergic Neurotransmitters

VitC is linked to brain signaling through the regulation of mono-aminergic neurotransmission by acting as co-factor of dopamine-β-hydroxylase in the conversion of dopamine to norepinephrine, and in enhancing the synthesis of serotonin and catecholamine precursors by supplying reducing equivalents for the tetra-hydrobiopterin-mediated hydroxylation of tryptophan and tyrosine [180,181,182,183]. Alongside increased MDA and protein carbonyls in the brain cortex, scorbutic *gulo*^−/−^ mice have reduced levels of dopamine and serotonin metabolites (3,4-dihydrophenylacetic acid (DOPAC), homovanilinic acid (HVA), 3-methoxytyramine (3-MT) and 5-hydroxyindoleacetic acid (5-HIAA)) [180]. The mice displayed reduced locomotor activity, grip strength and performance in maze-derived behavioral trials compared to wild-type and vitC supplemented *gulo*^−/−^ controls. Differences in behavior were eradicated following vitC repletion, supporting a direct effect of vitC on neuronal signaling and subsequent function [180]. Young guinea pigs with long-term (8 weeks) non-scorbutic vitC deficiency performed significantly poorer in the Morris water maze compared to controls and displayed a rise in the 5-HIAA:5-Hydroxytryptamine ratio in the hippocampus, indicating an imbalance of metabolites [184,185]. The recorded changes in behavior in vitC deficient groups may therefore be linked to disruptions in catecholamine signaling.

#### 4.3.2. Glutamate Signaling

A concentration-dependent relationship between glutamate and ASC in striatum and hippocampal regions (cornu ammonis 1,3 (CA1,3) and dentate gyrus (DG)) has been shown in vivo in rats, highlighting a dynamic interplay between glutamate signaling and ASC fluctuation in the brain, with putative effects on behavioral responses [186,187,188]. Upon release, glutamate can be taken up by astrocytes, converted to glutamine and released for neuronal uptake [63,189,190]. The uptake of glutamate in astrocytes prompts ASC efflux possibly through induced cellular swelling and volume-sensitive anion channels, releasing ASC, e.g., to diminish glutamate-induced oxidative damage [60,63]. Glutamate-induced ASC efflux in cultured neuroblastoma cells supports that glutamate uptake in neurons may also promote ASC efflux, likely through the involvement of volume-sensitive anion channels [61]. Failure to clear glutamate can induce neuronal excitotoxicity and oxidative damage through excessive stimulation of the N-*methyl*-D-*aspartate* (NDMA) receptor [93,190,191]. Glutamate excitotoxicity is associated with neuronal decay in hypoxia-ischemic injury in neonate animal models and is also likely involved in hypoxia-induced brain damage in infants [192,193]. Low brain ASC altered glutamate clearance and increased oxidative stress and sensitivity for seizure-induction as well as cognitive decline in mice models of Alzheimer’s Disease (*svct2*^+/−^-Amyloid precursor protein/Presenelin 1 (APP/PSEN1) mice and *gulo*^−/−^ APP/PSEN1 mice), linking vitC deficiency to dysregulation of glutamate and concurrent functional consequences [190,194].

Apart from a direct role in excitatory neurotransmission, glutamate is associated with development and maturation of the brain. Glutamate promotes neurogenesis by increasing proliferation of progenitor cells and indirectly by increasing growth factors such as brain-derived neurotrophic factor (BDNF) and insulin-derived growth factor 1 [195,196]. In addition, glutamate-induced synaptic Ca^2+^ influx reduces dendritic outgrowths and increases synaptogenesis, thereby regulating neuronal growth and synaptic plasticity [196,197].

In addition, ASC has also been linked to the modulation of the Gamma amino-butyric acid receptor subunit A (GABA_A_) receptor and subsequent potentiation of GABA_A_-mediated signaling in the CNS [198,199]. A role of ASC in ameliorating GABA and NMDA receptor dysregulation in depression has been suggested [199,200]. Though vitC deficiency may well be a factor in depression, clinical studies are few and often differ significantly in experimental design, analytical methodology and outcome measures, limiting comparison. A putative role of vitC in neuropsychiatric disorders has been reviewed in [201,202].

## 5. Effects of Vitamin C Deficiency on Brain Development

Malnutrition has been linked to negative effects in the brain and potential long-term consequences including reduced cognitive performance in children [203,204,205,206]. During embryogenesis and fetal development, signaling cues regulate events to proceed according to specific time-points at which a given process is initiated/completed. This makes specific cellular populations particularly vulnerable during these programmed events, and insults are often irreversible as induced damages to developing cells may compromise further progression [204]. Timing of developmental events include pre- and postnatal time-points, but whereas insults during early development may induce lasting changes, insults later in postnatal life may be less critical as the brain at this stage may have developed mechanisms—such as synaptic plasticity—to compensate for or even revert induced damage [204].

As studies of brain development in humans are extremely limited due to obvious ethical issues, experimental animal models constitute an important—if not the only—source of data. In this aspect, the guinea pig has distinct qualities compared to other rodents as the placental nutrient transfer resembles that of humans and controls the nutritional supply to the fetus during the main period of brain growth and myelinization [207,208,209,210,211]. Moreover, guinea pigs are born precocial and can be weaned at an early age allowing for independent interventions in young pups. A cautious comparison suggests that the brain neurogenesis in newborn guinea pigs resembles that of a 5-month-old infant, and that total brain neurogenesis equivalent to a human newborn is reached around gestational day (GD) 50 in guinea pigs compared to postnatal day (PD) 10 in mice and rats [212,213]. Importantly, extrapolating brain development stages from experimental models to humans must be viewed with significant translational limitations in mind.

### 5.1. Prenatal Effects of Vitamin C Deficiency

The extensive cellular metabolic activity of the growing fetus induces high levels of ROS, leading to oxidative stress and lipid peroxidation during pregnancy [214,215,216]. Combined with an immature antioxidative defense, this suggests that developing offspring may be particularly sensitive to reductions in antioxidant supply and hence are vulnerable to adverse effects of vitC deficiency [123,217].

#### 5.1.1. Fetal Vitamin C Levels

During gestation, vitC is transported across the placenta from mother to offspring through SVCT2-mediated transport [26,38,218]. In humans and guinea pigs, the fetus depends exclusively on an exogenous (maternal) supply, whereas most other mammals begin vitC synthesis late in gestation—in mice and rats around day 18 [7]. As the term approaches, maternal plasma concentrations decline while newborn infants display higher plasma vitC than their mothers [16,219,220]. This is also observed in guinea pigs, where newborn pups (PD7) show twice as high plasma ASC compared to dams [221]. At GD45, plasma ASC in fetal guinea pigs was almost three times that of maternal plasma (149 vs. 46 μM,) declining slightly towards term at GD56 (76 vs. 39 μM; guinea pig term is around GD60–65) [222]. Brain ASC levels were also significantly higher at GD45 compared to GD56 and postnatal levels [221,222,223].

The high ASC level in the GD45 guinea pig brain coalesces with the peak of overall brain growth measured as brain weight increase relative to the adult brain weight (“the brain growth spurt”) [224]. In humans, this occurs shortly before and in the first months after birth, whereas altricial species have a profound postnatal developmental phase; in mice and rats, the brain growth spurt peak is 1–2 weeks after birth [224]. The total brain weight is a crude method of developmental staging and does not directly reflect specific cell populations nor developmental key events such as glia formation, myelinization, synapse formation and the sequential neurogenesis in brain areas and regions. Still, the brain growth spurt reflects a time-point in which the brain is at its highest expansion rate and may be more vulnerable to insults—e.g., oxidative stress evoked damage [225].

#### 5.1.2. Neuronal Consequences

Weak capillary walls leading to petechial brain hemorrhages and subsequent loss of neuronal tissue in the cortex and brain stem is likely a primary cause of *svct2*^−/−^ mice dying immediately after birth [26,27]. However, terminal deoxynucleotidyl transferase dUTP nick end labelling (TUNEL) and isoketal positive staining were not limited to focal areas of bleeding, underlining that vitC depletion-induced lipid oxidation and apoptosis in the brain was not confined to areas of hemorrhagic hypoxia [27]. The very low brain ASC levels in *svct2*^−/−^ embryos at days 18.5–19.5 reduced cortical dopamine, norepinephrine and tyrosine hydroxylase, with no apparent effect on serotonin metabolism [181]. *Svct2*-overexpression in mice embryos significantly increased cortical levels of dopamine, DOPAC, serotonin and 5-hydroxyindole acetic acid, confirming SVCT2-mediated ASC transport to the brain cortex as instrumental in neurotransmitter synthesis during development [181]. In agreement with this, immuno-fluorescent quantification show marked decreases in tyrosine hydroxylase and 5-hydroxymethylation positive dopaminergic neurons and decreases in H3K27m3 positive dopaminergic neurons from *svct2*^−/−^ mouse embryos compared to *svct2*^+/−^ and *svct2*^+/+^ counterparts, indicating a direct effect of vitC depletion on developing dopaminergic neurons and also in DNA and histone methylation status [163].

In newborn *gulo*^−/−^ pups with vitC depletion during the last 2 weeks of gestation, brain hemorrhages within the parenchyma were evident, confirming weakening of brain capillaries [226]. In addition, lipid peroxidation and redox imbalance was recorded (increased MDA, 8-isoprostane and GSH:GSSG (oxidized glutathione)), and alterations in neuronal proliferation, maturation and cellular organization of the hippocampus and cerebellum were evident—e.g., increased staining of neuronal nuclei marker (NeuN) (and not glia marker (glia fibrillary acidic protein (GFAP)) in the hippocampus, abnormal fissure formation and reduced dendrite formation of Purkinje cells in the cerebellum compared to controls. Expression of BDNF and glial-derived neurotrophic factor was reduced in the brain, further supporting vitC deficiency-induced alterations in brain cell growth and structural development [226].

No difference at GD45 or 56 in hippocampal volume (both total and between subdivisions CA1–3 and DG) or β-tubulin isotype III staining in the *stratum lucidum* could be attributed to chronic vitC deficiency in guinea pigs [227]. However, brain MDA and the oxidative stress marker superoxide dismutase (SOD) was increased, confirming increased lipid perioxidation and redox imbalance with brain vitC levels of around 25–30% of controls [222,223]. In developing rat pups exposed to lead-induced toxicity in utero, ASC supplementation of dams (100 mg/kg body weight) improved SOD levels and cerebellar Purkinje cell morphology, synaptophysin expression and axonal myelinisation, linking vitC to a protective effect on oxidative stress-induced impaired neuronal development [228].

#### 5.1.3. Effect of Prenatal vitC Deficiency on Offspring Growth

*Svct2* knock-out (^−/−^) decreased numbers of both ^+/−^ and ^−/−^ offspring, diverting from a Mendelian ratio [27]. In pregnant *gulo*^−/−^ mice, vitC depletion significantly reduced numbers of live born pups, and in SMP30/GNL^−/−^ mice depletion led to low conception rates or early embryonic death, whereas deficiency (severe; tissue levels of around 10% of controls) increased perinatal deaths and severe organ malformations [226,229]. Female mice embryos (E13.5) derived from vitC depleted *gulo*^−/−^ dams displayed aberrant TET1 activity and deviated DNA demethylation in germ cell lines and reduced 5-hydroxymethylcytosine levels in embryonic brain and liver, suggesting that vitC deficiency can have a significant effect on TET1-associated embryonic DNA demethylation with negative consequences for development [161]. Moreover, prenatal vitC depletion in resulted in reduced oocyte formation (PD7) and decreased fecundity in first generation mating in *gulo*^−/−^ mice, even though vitC supplementation was re-instated from E13.5 onwards [161].

In vitC deficient guinea pigs (non-scorbutic) dams, body weight gain was reduced but litter size did not differ from controls at GD45, 56 or live-born to term (GD60–65) [74,222]. A significant reduction in placental and fetal body weight at GD45, indicated compromised fetal growth at this particular time-point [222]. Induced intrauterine growth restriction at midterm (GD30) has been shown to reduce cerebellar volume and neuronal number, and volume, neuronal numbers, dendrite formation and branching in the hippocampus in guinea pig offspring [230,231,232,233]. Alterations in white matter volume and myelinization in fetal guinea pigs subjected to intrauterine growth restriction has also been reported, but this appears to be restored in early adulthood [210,234]. In addition, deficient dams maintained a low vitC plasma level throughout and offspring did not increase concentrations at term, thereby deviating from the normal, physiological concourse of fetal vitC distribution [221,222]. An overview of the principal findings of prenatal vitC deficiency in experimental models is provided in Table 1.

#### 5.1.4. Clinical Studies

In humans, vitC has been suggested to be protective of some types of neural tube defects, but data on potential negative effects of vitC deficiency on embryonic and fetal development are lacking [235,236]. Reports from Brazil (*n* = 127 and 117 enrolled participants) show that almost 30% of parturient mothers were vitC deficient with plasma concentrations below 22.7 μM [16,17,237]. Habits of smoking and alcohol consumption significantly decreased vitC concentrations in umbilical cord samples, and a low maternal vitC status was reflected in breastfed infants [16,237]. A larger Aberdeen cohort (*n* = 1109) reported that 3% of enrolled women displayed vitC levels below 17 μM in early pregnancy (≥20 wks of gestation) and 4% at delivery, prevalence varying with smoking and educational status [219].

In a British cohort (*n* = 963), a low vitC intake during early pregnancy was associated with a lower birth weight in newborn children; however, circulating vitC levels were not reported, preventing a direct correlation to plasma concentration [238,239]. In agreement with this, a study in Korean women (*n* = 217) showed an association between a low maternal serum vitC in the second trimester and decreased neonatal weight and body length in infants born to term; a 1 μg/mL serum vitC increase leading to 0.17 cm more in infant body length, suggesting vitC as a key factor for optimal fetal growth [240]. This is supported by a recent report from The Korean Mothers and Children’s Environmental Health (MOCEH) cohort (*n* = 1138), in which a maternal diet low in vitC intake was found to be associated with decreased birth length and a reduced infant body weight from birth to 6 months of age [241].

In humans, intrauterine growth restriction and a low birth weight is linked to increased oxidative stress, neuro-inflammation, perinatal mortality and lasting deficits in learning and memory in children and adolescents [242,243,244,245,246,247]. In addition, low vitC during pregnancy is linked to increased preterm births, pre-eclampsia and increased placental apoptosis; however, reports of a beneficial effect of antioxidant supplementation on these outcomes are currently conflicting [239,248,249,250,251,252,253]. None of the included studies reported any clinically evident symptoms of scurvy, underlining that low vitC levels go undetected.

Collectively, the above findings point to serious effects of prenatal vitC deficiency on developing offspring. These include reduced conception rates and reductions in fetal growth, likely reflecting consequences of a suboptimal nutrition during pregnancy. In the brain, the induced alterations progress with the severity of deficiency, vitC depletion, leading to detrimental changes in the brain, whereas more moderate states of deficiency do not appear to induce the same degree of damage. However, putative functional consequences of a lack of sufficient vitC in utero will not be detected until after birth and often not before some degree of motor and cognitive development has been reached. At this point, damages may be irreversible and repletion may prove futile.

### 5.2. Postnatal Effects of Vitamin C Deficiency

#### 5.2.1. Perinatal Period and Early Life

With the first breath of air, a newborn must adapt to extreme changes outside the womb’s protective environment, such as increased oxygen concentrations, the dependency on an oral nutrient supply, and extensive growth combined with high cellular energy demands leading to ROS formation. The yet immature antioxidant system renders newborns prone to redox imbalance, potentially leading to free radical-induced toxicity and subsequent cell damage [254,255]. VitC is the primary antioxidant source in breastmilk and reflects maternal vitC status until saturation is reached, with mothers conveying a low vitC status to their infants [256,257,258]. A 1981 survey of Finnish parturient women (*n* = 200) reported 6% to be vitC deficient (plasma vitC below 11.3 μM), and studies from the U.S. and Brazil have reported low vitC status/hypovitaminosis C (plasma vitC below 28.4 or 22.7 μM, respectively) in up to 25–30% of parturient women, suggesting that vitC deficiency is not uncommon during pregnancy [17,258,259]. The Korean Ewha Birth and Growth cohort reported an association between a maternal vitC level below the 75 percentile and a decreased infant growth extending from birth until 36 months of age, indicating that effects may extend well into postnatal life [260].

#### 5.2.2. Lipid Peroxidation

In weanling guinea pigs, vitC depletion more than doubled the ascorbate oxidation ratio (ASC:DHA) and increased MDA and DNA repair mechanisms compared to non-deficient counterparts [24]. Blocked ASC transport to the brain in newborn *svct2*^−/−^ mice pups increased levels of F_2_-isoprostanes and F_4_-neuroprostanes and underlines PUFA peroxidation as a direct consequence of vitC deprivation to the brain [27,261]. In newborn mice, brain ASC content decreases after birth and significantly pronounced decreases in deficient *gulo*^−/−^ pups [97,137,262]. Low ASC increased MDA levels in the cerebellum at PD10, whereas F_2_-isoprostanes and GSH increased in the cortex but not the cerebellum at PD18, showing that lipid peroxidation differs between brain regions and over time [137].

Increased GSH was also reported at PD21 in whole brain homogenates of ASC deficient *gulo^−/−^* pups with brain ASC of around 25–30% of wild-type controls, supporting a disrupted redox balance and suggesting GSH as a possible compensatory response to low brain ASC in newborns [262]. Newborn guinea pigs (PD2–7) subjected to persistent pre- and postnatal vitC deficiency did not exhibit any clinical signs of scurvy nor increased MDA, 8-F2-isoprostane or GSH in the brain cortex compared to non-deficient counterparts, even though brain vitC levels were reduced by 60% [221].

#### 5.2.3. Changes in Brain Structure and Function

However, prenatal vitC deficiency significantly reduced the volume of the hippocampus at PD10 compared to non-deficient controls regardless of postnatal vitC supplementation, indicating irreversible effects on hippocampal morphology [74]. Staining of hippocampal sections at PD10 and PD27 showed reduced cell proliferation in the granular layer, but an increase in proliferating cells in the subgranular zone at PD27, suggesting that postnatal cellular migration in hippocampal subdivisions was delayed due to prenatal vitC deficiency [74]. In guinea pigs, prenatal exposure to vitC deficiency resulted in reduced hippocampal volume until PD70 even though vitC levels and the brain MDA, GSH and ascorbate oxidation ratios were restored after birth, establishing that induced preperinatal or/and perinatal damage persists at least until reproductive maturity [74,79]. There was no reported effects on locomotion, and contrary to previously observed differences in spatial memory [185], the animals in this study mainly exhibited a random swim pattern in the Morris water maze irrespective of vitC status [74]. Whereas a spatial swim pattern is directed mainly to the platform quadrant of the maze—reflecting the animals’ ability to remember and apply visual cues—a random pattern is characterized by an absence of a preferred quadrant and does not target the platform area; hence, animals do not appear to apply or remember spatial cues when placed in the maze. The absence of a spatial swim pattern in almost all animals of this study therefore prevented the subsequent evaluation of spatial memory competence and hippocampal function between experimental groups, unfortunately limiting any conclusions regarding the functional effects of prenatal vitC deficiency in this study [74]. (A brief overview of main findings of vitC deficiency imposed postnatal effects is provided in Table 2 and Table 3).

#### 5.2.4. Infants Born Preterm

A premature birth may exacerbate the challenges faced after birth and is associated with increased oxidative stress, peroxidation of PUFAs and risk of neurological impairments such as learning disabilities and reduced sensory and motor functions, highlighting the sensitivity of the newborn brain towards adverse levels of oxidative stress and the potential induction of long-term consequences [255,267]. In addition, a lack of oxygen to the brain, e.g., due to neonatal hypoxia and/or ischemia, can inflict serious consequences; increased sensitivity to hypoxia-induced damage has been shown in the brain cortex and thalamus with glutamate excitoxicity as a key inducer of neuronal damage in neonates [192,193]. Interestingly, the distribution of injury differs between preterm and term newborns and emphasizes timing and the developmental stage as pivotal in the concourse of induced and putatively damaging effects [193,268,269,270].

Specifically for vitC, the physiological increase in fetal vitC towards term may not have been reached at delivery, leaving premature infants with a low vitC status. Baydas et al. reported significantly lower levels of vitC in umbilical cord blood from preterm compared to term infants, though maternal vitC levels did not differ (mean plasma concentration of around 70 μM) [220]. Breastmilk from mothers giving birth preterm differs in some aspects of composition, but redox properties and vitC were found to be largely preserved, albeit decreasing with the degree of prematurity [271,272].

Immaturity also compromises the intake and absorption of nutrients across the intestinal tract, in many cases leaving parenteral nutrition necessary. As parenteral nutrition is prone to spontaneous generation of peroxides when exposed to oxygen and ambient light, this constitutes a potential source of increased oxidative stress at infusion, thereby unintentionally contributing to increase the oxidative stress burden on the already challenged infant [254,273,274,275].

Unfortunately, vitC requirements of the preterm infant beyond avoiding scurvy are mainly unknown, rendering the assessment of “sufficient” vitC contents in parenteral nutrition difficult. In addition, vitC transport mechanisms changes during development and may consequently reduce or alter absorption and distribution to cells in immature newborns, further complicating translation between administered vitC and tissue levels [97,276].

The highly oxidative environment, increased risk of infections and inflammatory diseases compromised nutrition and a limited antioxidant defense places prematurely born infants in a self-propagating circle of potentially induced damage to the developing brain. Reduced hippocampal volume and reduced learning and memory ability was reported for 2-year old children born before week 32 of gestation [277]. A recent meta-analysis of the cognitive abilities in children born preterm discloses a significantly reduced IQ in children born very preterm (<32 weeks) compared to term counterparts [278]. Notably, though perinatal care had evolved and seemingly improved, the measured effects on cognitive outcomes had not improved across the 1990–2008 time-span [278].

Thus, the first part of life represents a period of dramatic change for the developing infant, also with regard to putative negative effects of vitC deficiency. Induced changes include increased oxidative stress and lipid peroxidation in the brain; however, these may not be a prerequisite for structural alterations. Infants born preterm represent a particularly vulnerable subgroup, in which antioxidant defenses are reduced in combination with several additional factors that may exacerbate damaging effects on the perinatal brain.

### 5.3. Vitamin C Deficiency in Young Life

Though uncommon compared to the historic prevalence, scurvy is still encountered in young children, and also in developed countries, due to, for example, restrictive eating habits or conditions [279]. Initial symptoms are diverse and unspecific (irritability, fatigue, reluctance to move), and signs may easily be overlooked or misinterpreted delaying diagnosis and subsequent treatment [144,279,280]. In humans, the first 2–3 years of life represent a time of extensive structural development and maturation of the brain, making this a period of increased sensitivity to insults and the “first 1000 days” of life an opportunity to reduce detrimental effects on the brain by, e.g., ensuring that nutritional needs are met [224,281,282,283].

#### 5.3.1. Redox Homeostasis

Young (PD18) vitC deficient *gulo*^−/−^ mice displayed increased brain GSH and F_2_-isoprostanes, but not MDA in the brain cortex [137]. In agreement with this, in weanling PD21 *gulo*^−/−^ mice, with brain ASC levels of around 30% of wild-type controls, GSH levels were increased; however, at PD60, no difference in GSH could be recorded despite consistently low brain ASC levels [262]. Reports of severely vitC deficient and depleted adult *gulo*^−/−^ mice show increased brain oxidative stress markers, MDA, 8-isoprostanes and GSH (and increased GSH:GSSG), and induced expression of pro-inflammatory cytokines, with no observed alterations in brain histology or reductions in working spatial memory, but decreased motor competence [180,226,263]. In 30-day-old SMP30/GNL^−/−^ mice, vitC depletion for 4 and 8 weeks (but not 2) significantly increased superoxide generation in ex vivo brain slices and stated findings of histologically evident cell death in the cerebellar cortex after 8 weeks of depletion (though data are not shown) [265]. At 8 weeks of depletion, animals displayed 30% reduction in body weight compared to controls, underlining the severity of depletion and the presence of a scorbutic state; hence, findings should be interpreted with this in mind. Though strains and the degree of the imposed vitC deficiency vary between studies, the above findings may indicate age-related differences in the response to vitC deficiency and potential functional effects—for example, increased lipid oxidation in older animals compared to newborns.

Severe vitC deficiency induced in 1-week-old guinea pig pups for 11 weeks (plasma concentration of 2.2 μM, resulting in a prescorbutic state) did not increase brain MDA or GSH levels, though vitC levels were less than one-third of the controls [184]. Markers of synaptic plasticity in the frontal cortex, hippocampus or striatum did not differ with degree of vitC deficiency (moderate vs. severe deficiency) and dendrite morphology of hippocampal CA1 was not affected in severely deficient animals [184]. Levels of neurotransmitters or spatial memory competence were not measured, preventing the assessment of functional effects.

In weanling guinea pigs subjected to vitC depletion after birth (PD2), brain MDA and SOD were increased; however, the degree of deficiency was more severe and, at an earlier time-point in development, potentially contributed to increased sensitivity due to higher levels of brain growth, reduced antioxidant capacity following birth and general immaturity, including the adaptation to independent nutrition [24,284]. In this regard, the studies represent two different scenarios with very different outcomes—one leading to clinical scurvy and the other one remaining clinically undetectable, while both result in negative changes in the young brain.

The absence of induced redox imbalance may also reflect a species-associated difference in response and/or compensatory mechanisms following vitC deficiency in mice vs. guinea pigs, possibly through evolutionary adaptation. Species differences such as the effective use of DHA as a vitC source and carnitine response during deficiency support that guinea pigs and humans have similar mechanisms for maintaining vitC homeostasis, whereas this may not be the case for vitC synthesizing species such as mice and rats [71,285]. How this may affect vitC levels in the brain remains to be determined.

#### 5.3.2. Changes in Brain Structure and Function

In 1-week-old guinea pigs, a chronic, non-scorbutic, vitC deficiency resulted in reduced spatial memory competence in the Morris water maze compared to controls at PD50 [185]. Stereological evaluation of the hippocampus revealed significantly fewer neurons in all three subdivisions (CA1, CA2–3 and dentate gyrus), linking postnatal vitC deficiency to reduced neuronal numbers and functional consequences in the brain [185]. Reflecting vitC intake, brain ASC levels were reduced to less than 50% and the ascorbate oxidation ratio increased in deficient animals compared to controls. There was no apparent effect on SOD, GSH or MDA levels in the brain, suggesting that the effects of deficiency could be due to mechanisms not directly associated with oxidative stress [185]. In agreement with an effect on more specific functions, reduced levels of synaptophysin and alterations in serotonin metabolites in the hippocampus of deficient animals suggested impaired neuronal signal transmission, potentially exacerbating the consequences of the lower neuronal numbers [266].

In *gulo*^−/−^ mice subjected to chronic postnatal vitC deficiency, behavioral tests (PD60–100) disclosed slight reductions in locomotor ability, and no effect on hippocampal learning ability in the Morris water maze; however, the effect on long-term spatial memory (retention test) was not assessed [262]. Alterations in pharmacologically induced functional responses supported an imbalance in the regulation of brain dopamine in vitC deficient animals [262]. In juvenile (4-week-old) *akr1a*^−/−^ mice, short-term vitC depletion (1 week) impaired spatial memory, whereas this was not the case in chronically vitC deficient young adult *akr1a*^−/−^ mice (12–13 weeks of age) despite lower brain ASC levels in adults vs. juveniles [264]. This may indicate that the juvenile hippocampus requires increased vitC levels during development of functional neuronal circuits, but also that, in *akr1a*^−/−^ mice, the developing hippocampus may be able to compensate for the impaired spatial function over time. Notably, *akr1a*^−/−^ mice display several additional deficits in addition to the reduced ability to synthesize ASC; findings should be interpreted with this in mind [70].

Despite extremely low ASC levels, scorbutic *gulo*^−/−^ mice were able to move voluntarily, indicating that the observed locomotor deficits were not caused exclusively by physical impairment and could include additional effects on neuronal signaling [180]. No differences in brain histology were reported; however, metabolites of dopamine and serotonin increased in the cortex, whereas only 5-HIAA decreased in the striatum, underlining differences in regional responses to severe vitC depletion [180]. Interestingly, social dominance behavior was reduced during depletion—before clinical symptoms of scurvy—possibly reflecting a depressive-like state, which did not improve once ASC supply was restored [180]. A brief overview of the main findings from experimental models is provided in Table 2 and Table 3.

#### 5.3.3. Vitamin C Status in Children

Reports of vitC status in children from different subpopulations and demographics are unfortunately scarce. The National Health and Nutrition Examination Survey (NHANES, U.S.) 2003–2004 reported a vitC status below 28 μM in almost 20% in the 6–19-year-old age group [15]. Compared to data from NHANESIII (1993–1994), the overall prevalence of vitC deficiency in children was reduced, likely illustrating an improvement in vitC status due to changes in eating habits [15]. Though showing a positive trend, the data emphasize that a significant part of children and adolescents may suffer from hypovitaminosis C and therefore be at risk of experiencing negative consequences of vitC deficiency [15,286]. Severe vitC deficiency (plasma <11.3 μM) was reported for almost one-third of 0–2-year-old Mexican children, with a mean prevalence of 23% in children <12 years old (*n* = 1815) [18]. The general prevalence of low vitC levels is increased in families of low socio-economic status and associated with risk factors such as smoking and obesity, which are also associated with low socioeconomic status, highlighting that selected subgroups are likely to be at increased risk of a deficient vitC status [15,18,287].

## 6. Potential Challenges When Evaluating Clinical Studies of Vitamin C

According to the reported prevalence of vitC deficiency in the general population, hypovitaminosis C may affect millions worldwide [20]. Moreover, vitC deficiency is likely more frequent during pregnancy and childhood, categorizing mothers-to-be and their children as subgroups of potentially increased risk of hypovitaminosis C or even severe deficiency.

Unfortunately, available reports of vitC status in newborns and children are few and most are decades old and may therefore not adequately reflect current population status. Studies during pregnancy and in infants/children are further challenged by relatively low sample sizes reducing power and are sensitive to selection bias—e.g., by sampling only from patients admitted for increased monitoring (high risk groups) such as pregnancy-associated complications (reviewed in [288]). More substantial reports on vitC status are available from the general adult population, but updated and valid data from large scale investigations of vitC status are few [20]. Unfortunately, the principle of the design of an epidemiological survey limits conclusions of any causal relationship between an isolated factor—in this case, vitC status—and concurrent disease, as additional factors are not controlled for [22]. This could, for example, accompany nutritional deficiencies, which could well affect outcome measures, thereby confounding conclusions.

A general point of criticism in clinical studies is also that the integrity of findings may be hampered by flaws in study design. For example, the absence of baseline vitC measurements and subsequent determination of vitC deficiency as a predefined inclusion criterion is unfortunately often the case particularly in older reports [12,289]. As increased vitC intake results in plasma saturation, supplementation of individuals already close to saturation will only lead to subtle effects at best. A lack of stratification for vitC status at inclusion may therefore mask the detection of potential benefits of supplementation in deficient groups. In addition, vitC transport may be subject to genetic variation, in turn affecting individual vitC levels and consequent responses to supplementation [43,290]. Application of qualitative data of vitC ingestion collected through patient recollections and self-reporting of diet composition can be prone to inaccuracies, limiting the value of information [12,291,292,293]. Moreover, clinical trials investigating putative effects of vitC supplementation often include combinations of antioxidants, commonly vitC and vitE, in variable dose regimes and during different intervention periods, complicating comparisons and preventing conclusions of isolated effects.

Lastly, differences in analytical methodology of vitC measurements can prevent meaningful comparisons between studies [21,22]. Specifically for vitC, sample preparation is crucial to avoid spontaneous oxidation and subsequent ASC deterioration, consequently leading to faulty conclusions of low vitC levels [294,295]. Other analytical methods may instead lead to overestimations of vitC in samples [294,296]. These risks of wrongfully estimating vitC levels naturally have serious implications for data integrity and must be carefully addressed when designing novel studies.

Thus, while clearly showing that vitC supplementation is safe, the clinical literature has not provided much relevant information on the potential benefit of supplementation to vitC deficient children. Most countries recommend a surplus intake of vitC during pregnancy (10–20 mg/day) and lactation (20–60 mg/day) to accommodate for increased maternal requirements [10]. For infants and young children, vitC reference intakes are commonly based on the estimated average vitC content and intake of breastmilk and approximated food content when applicable, whereas recommendations during childhood/adolescence are derived from the RDI for adults and adjusted for differences in body weight [10,25]. VitC deficiency during pregnancy and in infants and children should therefore be prevented if the guidelines from health authorities are followed.

However, guidelines may not apply or be followed by all. Children exposed to risk factors such as smoking or premature birth, or children in subgroups where vitC intake from fresh fruit and vegetables is low, e.g., in low income families or during seasonal changes, may potentially benefit from additional supplementation. In addition, single nucleotide polymorphisms of the SVCT allele have been suggested to affect transport capacity and subsequently vitC homeostasis [44]. The functional effects and population prevalence of such SVCT polymorphisms have not yet been established, but may render genotype as an important factor when identifying individuals of increased risk of vitC deficiency. Should this association prove to be true, a genotype-induced vitC deficiency could be explored in future study designs, in which individuals with SVCT polymorphisms might provide insights on the isolated effects of a life-long state of vitC deficiency [22].

## 7. Concluding Remarks

The reported findings confirms that long-term non-scorbutic vitC deficiency can lead to structural changes and functional impairments in the brain. These effects may not be immediately apparent, but instead manifest later as, e.g., dysfunctional signaling that in turn may compromise cellular development. Notably, even chronically low levels of vitC both during pregnancy and after birth did not cause symptoms of scurvy, emphasizing that hypovitaminosis C is very likely to go unnoticed.

The complexity of transport systems and intracellular recycling mechanisms complicates our ability to accurately extrapolate vitC plasma concentrations to tissue levels in animal models as well as in humans. The unique features of the guinea pig suggests that this animal model is superior to other rodent models of vitC deficiency, corroborating that findings may be of high translational value. However, to which degree the applied dose levels in guinea pigs can be translated to humans and how vitC deficiency may lead to functional consequences in the human brain remains difficult to predict. It is clear, however, that specific subgroups such as pregnant women, prematurely born children and families of low income and educational levels are at increased risk of deficiency. Delayed or impaired brain development would be an additional challenge to already vulnerable children, potentially limiting their ability to advance and reach their full learning potential.

## Figures and Tables

**Table 1:** Principal findings of prenatal vitamin C deficiency—findings from experimental animal models.

VitC	Species/*Strain*	Time-Point	Principal Findings	Ref
Depletion (to the brain)	Mice/*svct2*^−/−^	E18.5–19.5 Term	Neonatal deaths. Petechial bleedings on brain surface an in parenchyma, reflecting weakened capillary walls. Increased lipid peroxidation (isoketals). Neuronal apoptosis in cerebral cortex and brain stem. Altered regulation of norepinephrine and dopamine and reduced dopaminergic neurons (decreased tyrosine kinase positive neurons). Aberrant DNA and histone methylation status.	[26,27,181]
Depletion	Mice/*gulo*^−/−^	Term	Neonatal deaths. Petechial bleedings in brain parenchyma. Increased lipid peroxidation (MDA, 8-isoprostane), redox imbalance (increased GSH:GSSG and NO). Deviated structural development in cerebral cortex, hippocampus and cerebellum. Reduced BDNF and GDNF.	[226]
Deficiency	Mice/*gulo*^−/−^	Term (E20)	Increased lipid peroxidation (MDA) in cerebellum but not cortex.	[137]
Deficiency	Guinea pig/*Dunkin Hartley*	GD45 and GD 56	Increased lipid peroxidation (MDA) at GD 56 not 45. Redox imbalance marker (SOD) was increased in both GD45 and 56. No effect on hippocampal volume or β-tubulin III in hippocampal stratum lucidum. Transitional growth reduction reported for GD45.	[74,227]

BDNF: Brain-derived neurotrophic factor; deficiency: low vitC supplementation depletion: no vitC supplementation; E: embryonic day; GD: gestational day; GDNF: glia-derived neurotrophic factor; GSH: glutathione; GSSG: oxidized glutathione; MDA: malondialdehyde; NO: nitric oxide; SOD: superoxide dismutase.

**Table 2:** Principal findings of postnatal vitamin C deficiency in the brain—findings from murine models.

VitC	*Strain*	Time-Point	Principal Findings	Ref
Deficiency	*gulo* ^−/−^	PD1	No reported change in lipid peroxidation.	[137]
		PD10 PD18	Increased lipid peroxidation (MDA) in cerebellum, not cortex.	
			Increased lipid peroxidation (F_2_-isoprostanes) in cortex not cerebellum. Increased redox imbalance (GSH) in cortex. Possible increase in GFAP stained cells (astrocytes) albeit not quantified. No functional effects on locomotion, agility or strength were detected.	
Depleted	*gulo* ^−/−^	PD21	Increased redox imbalance (GSH).	[262]
Deficient		PD60–100	No redox imbalance. Reduced locomotion but no effect on spatial learning (MWM). Spatial memory was not assessed. Enhanced response to dopaminergic agonist indicating deviated regulation of dopaminergic signaling.	
Depletion	*gulo* ^−/−^	Young adults (20 gr)	Increased lipid peroxidation (MDA) and increased protein carbonyls in cortex. Decreased dopamine and serotonin metabolites in cortex and striatum. Locomotor deficits and reduced social dominance.	[180]
Depletion	*gulo* ^−/−^	4 wks–8 wks	Increased lipid peroxidation (MDA) in cortex, not cerebellum.	[97]
Deficiency		4 wks–8 wks	Increased lipid peroxidation (MDA) in cortex, not cerebellum.	
Deficient	*gulo* ^−/−^	6–18 wks old	Increased F_4_-neuroprostanes (also in vitC supplemented *gulo*^−/−^ counterparts). Reduced sensimotory competence, most significant in deficient *gulo*^−/−^. Memory and cognition was not affected.	[263]
Depletion (acute)	*akr1* ^−/−^	Juvenile (5 wks old–1 wk deplet.)	No apparent redox imbalance. No recorded changes in hippocampal histology (*n* = 2). Reduced spatial memory competence. No effect on neurotransmitters (dopamine, norepinephrine, glutamic acid, GABA, acetylcholine and selected metabolites).	[264]
Deficiency (long term)		Adult (12–13 wks)	No effect on spatial memory competence.	
Depletion	SMP30/ GNL^−/−^	PD30- 2,4,8 wks depletion	4- and 8-wk depletion increased superoxide production ex vivo; reduced cells in cerebellar cortex after 8-wk depletion (though data not shown). No effect on SOD expression or activity.	[265]

Deficiency: low vitC supplementation; depletion: no vitC supplementation; GABA: gamma aminobutyric acid; GFAP: glial fibrillary acidic protein; GSH: glutathione; MDA: malondialdehyde; PD: postnatal day; SOD: superoxide dismutase; wks: weeks.

**Table 3:** Principal findings of postnatal vitamin C deficiency in the brain—findings from guinea pigs.

VitC	*Strain*	Time-Point	Principal Findings	Ref
Depletion	*Dunkin Hartley*	PD2–3 wks	Increased lipid peroxidation (MDA), increased protein carbonyls, induced DNA-based excision.	[24]
Severe deficiency	*Dunkin Hartley*	PD7–11 wks	No effects on the investigated hippocampal structures or synaptic plasticity markers and BDNF in cortex, hippocampus or striatum.	[184]
Deficiency			No additional apparent differences compared to severe deficiency.	
Pre- and postnatal deficiency	*Dunkin Hartley*	PD2–7	No effect on lipid peroxidation (MDA, 8-F_2_-isoprostane); GSH not different.	[221]
		PD10	Reduced hippocampal volume and reduced proliferation in hippocampal granular layer.	[74]
		PD27	Reduced hippocampal volume and increased proliferation in granular layer and subgranluar zones.	
		PD70	Increased lipid peroxidation (MDA). Hippocampal volume reduction. Persistent decrease in hippocampal volume despite vitC repletion after birth.	[74,79]
Deficiency	*Dunkin Hartley*	PD7–9 wks	No effect on lipid peroxidation (MDA) or redox markers (SOD, GSH). Reduced neuron numbers in hippocampus. Deviated serotonin metabolites and reduced synaptophysin. Reduced spatial memory competence.	[185,266]

BDNF: brain-derived neurotrophic factor; deficiency: low vitC supplementation; depletion: no vitC supplementation; GSH: glutathione; MDA: malondialdehyde; PD: postnatal day; SOD: superoxide dismutase; wks: weeks.
